# Evaluation of 3D T1-weighted spoiled gradient echo MR image quality using artificial intelligence image reconstruction techniques in the pediatric brain

**DOI:** 10.1007/s00234-024-03417-9

**Published:** 2024-07-05

**Authors:** Usha D. Nagaraj, Jonathan R. Dillman, Jean A. Tkach, Joshua S. Greer, James L. Leach

**Affiliations:** 1 Department of Radiology and Medical Imaging, Cincinnati Children’s Hospital Medical Center, 3333 Burnet Avenue, Cincinnati, OH 45229-3026 USA; 2 Department of Radiology, University of Cincinnati College of Medicine, Cincinnati, OH USA; 3 Philips Healthcare, Cincinnati, OH USA

**Keywords:** Artificial intelligence, Brain MRI, Pediatrics, Compressed sensing

## Abstract

**Purpose:**

To assess image quality and diagnostic confidence of 3D T1-weighted spoiled gradient echo (SPGR) MRI using artificial intelligence (AI) reconstruction.

**Materials and methods:**

This prospective, IRB-approved study enrolled 50 pediatric patients (mean age = 11.8 ± 3.1 years) undergoing clinical brain MRI. In addition to standard of care (SOC) compressed SENSE (CS = 2.5), 3D T1-weighted SPGR images were obtained with higher CS acceleration factors (5 and 8) to evaluate the ability of AI reconstruction to improve image quality and reduce scan time. Images were reviewed independently on dedicated research PACS workstations by two neuroradiologists. Quantitative analysis of signal intensities to calculate apparent grey and white matter signal to noise (aSNR) and grey-white matter apparent contrast to noise ratios (aCNR) was performed.

**Results:**

AI improved overall image quality compared to standard CS reconstruction in 35% (35/100) of evaluations in CS = 2.5 (average scan time = 221 ± 6.9 s), 100% (46/46) of CS = 5 (average scan time = 113.3 ± 4.6 s) and 94% (47/50) of CS = 8 (average scan time = 74.1 ± 0.01 s). Quantitative analysis revealed significantly higher grey matter aSNR, white matter aSNR and grey-white matter aCNR with AI reconstruction compared to standard reconstruction for CS 5 and 8 (all p-values < 0.001), however not for CS 2.5.

**Conclusions:**

AI reconstruction improved overall image quality and gray-white matter qualitative and quantitative aSNR and aCNR in highly accelerated (CS = 5 and 8) 3D T1W SPGR images in the majority of pediatric patients.

**Supplementary Information:**

The online version contains supplementary material available at 10.1007/s00234-024-03417-9.

## Introduction

Magnetic resonance (MR) imaging offers the most comprehensive structural evaluation of the brain compared to all other available imaging modalities in clinical practice and has become an essential component in the evaluation of children with neurological impairments. MR imaging, however, requires relatively long image acquisition times and is sensitive to motion artifacts which are common in children. This makes techniques aimed at shortening scan time important in order to increase exam success rates, facilitate clinical throughput, improve patient satisfaction, and avoid sedation or general anesthesia [1]. MRI scan time reduction techniques such as parallel imaging and compressed sensing (CS) have been developed for this purpose, however often come at a cost of decreased signal-to-noise ratio (SNR) and/or spatial resolution [2].

Artificial intelligence (AI) methods allow for improved MR image quality by applying deep learning-based reconstruction schemes to fully or under-sampled k-space data resulting in improved or preserved image quality in equivalent or reduced acquisition time [3]. However, their use in clinical practice, and in pediatric neuroimaging in particular, has yet to be adequately explored. Such evaluation is needed as these algorithms are trained mostly, and typically even entirely, on adult datasets [4].

The purpose of this study is to assess the impact of what is now a U.S. FDA-approved AI MR image reconstruction algorithm on image quality and diagnostic confidence of pediatric brain 3D T1-weighted spoiled gradient echo (SPGR) MR imaging at standard clinical and accelerated compressed SENSE (CS) for potential clinical use in the pediatric population.

## Materials and methods

This prospective, IRB-approved study included 50 pediatric patients undergoing routine, clinical brain MRI examinations without intravenous contrast at Cincinnati Children’s Hospital Medical Center enrolled between July 2022 and January 2023. Exclusion criteria included age less than two years, need for imaging under general anesthesia or sedation, and history of neurologic surgery and/or the presence of intracranial implants. Patients were recruited by phone call from a study coordinator prior to the date of the scheduled clinical examination. Informed consent was obtained from all participants, and informed assent was obtained, as appropriate.

### Scanning parameters

All imaging was performed on the same 1.5T MRI scanner (Ingenia; Philips Healthcare; Best, the Netherlands) using the body coil transmit and a 15 channel receive only head coil. Sagittal 3D T1-weighted SPGR (TR = 8.49 ms, TE = 4.6 ms, 1 mm contiguous interleaved slices; CS = 2.5) was performed as part of the clinical protocol in all enrolled patients. In the first 26 patients enrolled, additional sagittal 3D T1-weighted SPGR images were obtained with CS of 8. In the last 24 patients enrolled, additional sagittal 3D T1-weighted SPGR images were obtained with CS of 5. All other imaging parameters were unchanged. All images were reconstructed by both standard CS (medium denoising) image reconstruction and with the prototype AI reconstruction provided by the vendor. The AI network is based on an Adaptive-CS-Net, and is now an FDA-approved Philips product known as SmartSpeed [3, 5]. Additional details on the architecture have been previously published [6]. The “strong” AI denoising algorithm was selected from a 4-point scale: weak, medium, strong, and maximum.

### Image evaluation

**Fig. 1:**
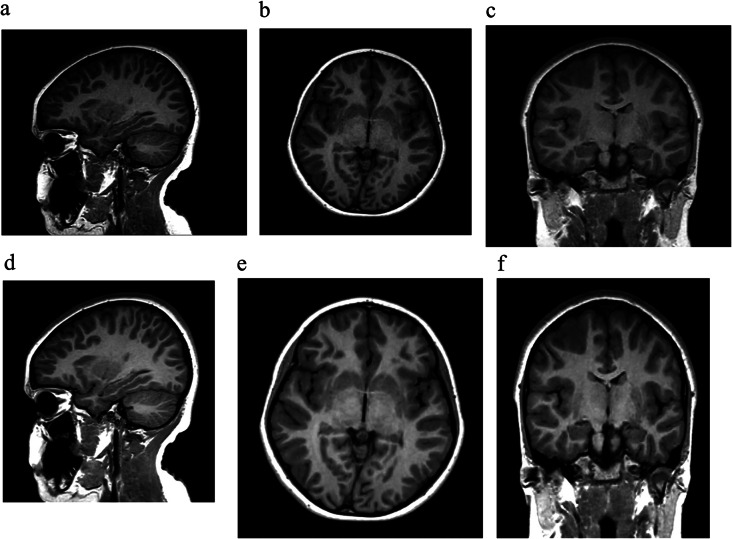
(**a**-**f**): Sagittal 3D T1-weighted SPGR CS = 2.5 with axial and coronal reformats from the clinical protocol was performed with standard (**a**-**c**) and AI (**d**-**f**) reconstruction, with little perceived difference in overall image quality

**Fig. 2:**
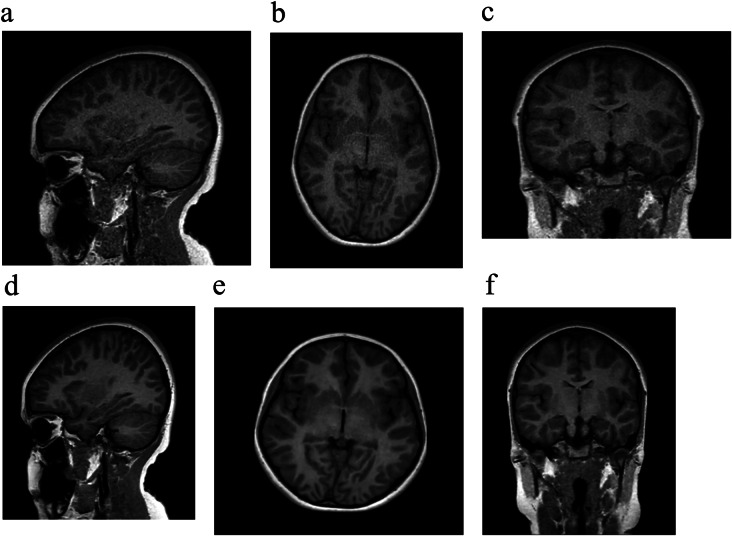
(**a**-**f**): Sagittal 3D T1-weighted SPGR CS = 8 with axial and coronal reformats in the same patient as Fig. 1 was performed with standard (**a**-**c**) and AI (**d**-**f**) reconstruction with improved overall image quality on the AI sequences marked by increased SNR and image sharpness

**Fig. 3:**
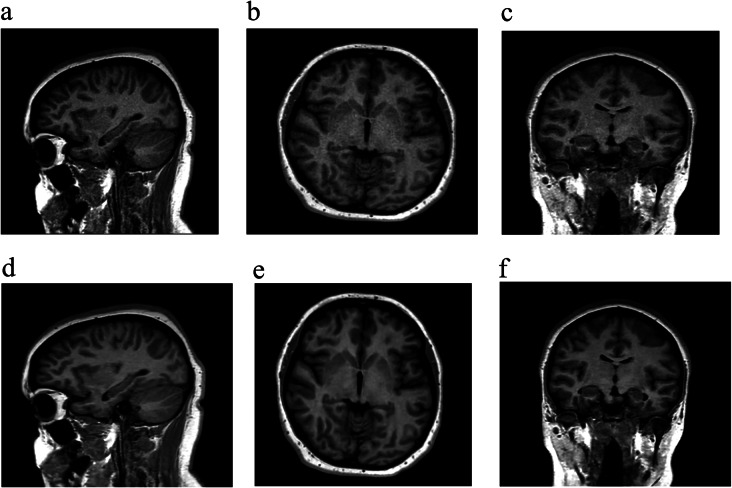
(**a**-**f**): Sagittal 3D T1-weighted SPGR CS = 5 with axial and coronal reformats in a different patient with standard (**a**-**c**) and AI (**d**-**f**) reconstruction also demonstrates improved image quality with AI

All 3D T1-weighted SPGR images were reviewed independently on a dedicated research PACS workstation by two fellowship trained attending pediatric neuroradiologists (UDN and JLL), who were blinded to patient information and clinical data during the review process (Figs. 1, 2 and 3). The imaging data sets were evaluated side by side without blinding to the sequences themselves, so the radiologists were able to determine the CS value and reconstruction method for each. AI images were evaluated on a 3-point scale (poor, sufficient, excellent) for overall image quality, subjective assessment of SNR, general noise/artifacts, and diagnostic preference (Supplementary Table A). Each sequence was also rated on a 3-point scale to indicate whether AI increased, decreased, or had no effect on image quality compared to the standard CS reconstruction. Additional imaging features were assessed and compared between the standard CS and AI reconstruction images and included CSF artifacts, motion artifacts, susceptibility artifacts, grey-white matter differentiation, image sharpness, flow void visualization and extracranial structure evaluation (Supplementary Table A). The presence of pathology, normal variants, or lack thereof was also recorded. Study interpretations were subsequently compared to the official clinical reports to exclude the possibility of overlooked clinically relevant pathology.

Scan duration was extracted for each sequence from the DICOM header. Quantitative analysis was performed using Philips IntelliSpace Portal (Version 10.1, Philips Healthcare). Circular regions of interest (ROIs) were manually placed by the neuroradiologist (UDN) in the head of the right caudate nucleus and right frontal white matter, and the mean and standard deviation of the signal within each ROI were used to calculate apparent signal to noise (aSNR) grey matter, aSNR white matter, and grey-white matter apparent contrast to noise ratios (aCNR) [7].

Statistical analysis was performed using Excel (2021, Microsoft) spreadsheet mathematic functions including mean values, standard deviations, value count and *t*-test results. P-values less than 0.05 were considered statistically significant. Descriptive statistical analyses of the independent reviews were performed. We did not use Kappa coefficients due to unbalanced observation across scores.

## Results

### Summary of cohort

A total of 50 patients (mean age = 11.8 ± 3.1 years, 42% (21/50) male) undergoing clinical brain MRI were enrolled. 3D T1-weighted SPGR CS = 2.5 (average scan time = 221 ± 6.9 s) scans were successfully performed in all study participants. The first 26 patients enrolled had an additional 3D T1-weighted SPGR CS = 8 (average scan time = 74.1 ± 0.01 s) scan attempted, of which 25 were successfully performed. The last 24 patients enrolled had an additional 3D T1-weighted SPGR CS = 5 (average scan time = 113.3 ± 4.6 s) scan attempted, of which 24 were successfully performed, although standard CS reconstruction images were not available for the 3D T1 SPGR CS = 5 at time of analysis in 1 patient due to an error in image to transfer to research PACS.

58% (29/50) of the patient exams were interpreted as normal. Abnormalities identified included foci of presumed gliosis (*n* = 6), Chiari I (*n* = 2), mild ventriculomegaly (*n* = 1), arachnoid cysts (*n* = 1), dolichocephaly (*n* = 1), cystic encephalomalacia (*n* = 1), tuberous sclerosis (*n* = 1) and a right thalamic mass (*n* = 1). Normal variants included mega cisterna magna (*n* = 2), cerebellar developmental venous anomaly (*n* = 1), enlarged perivascular spaces (*n* = 1), and cerebellar ectopia not meeting imaging criteria for Chiari I (*n* = 1).

### 3D T1-weighted SPGR CS = 2.5 (Standard of Care Clinical Protocol)

Overall image quality, degree of noise/artifacts and diagnostic confidence were rated as excellent in 81% (81/100), 79% (79/100) and 84% (84/100) respectively on 3D T1-weighted SPGR CS = 2.5 with AI **(**Figs. 1 and 4a). Compared to standard CS reconstruction images, AI reconstruction improved overall image quality, subjective SNR and diagnostic confidence in 35% (35/100), 40% (40/100) and 35% (35/100) respectively. When comparing AI and standard CS reconstruction the degree of CSF artifacts, motion artifacts, susceptibility artifacts, flow void visualization and extra-cranial structure visualization were felt to be the same in 100% of exams. These and other data are summarized in Table 1. Quantitative analysis of signal intensities (mean ROI area = 60 ± 11.9 mm^2^) revealed no significant difference in white matter aSNR (32.9 ± 11.7 vs. 29.5 ± 9.7, *p* = 0.12) and grey matter aSNR (23.4 ± 6.4 vs. 21.1 ± 4.7, *p* = 0.05) with AI when compared to standard CS reconstruction, respectively. The grey-white matter aCNR was also not significantly different with AI (5.1 ± 1.4) compared to standard CS reconstruction (4.6 ± 1.2, *p* = 0.07).

**Table 1:** T1-weighted SPGR CS = 2.5 AI vs. standard CS reconstruction

T1 CS 2.5 AI vs. Standard CS Reconstruction	Reviewer 1 (*n* = 50)	Reviewer 2 (*n* = 50)	% Concordance
Overall Image Quality	100% (50/50) Same	70% (35/50) Better 30% (15/50) Same	30% (15/50)
Subjective SNR	4% (2/50) Better 96% (48/50) Same	76% (38/50) Better 24% (12/50) Same	28% (14/50)
Diagnostic Preference	100% (50/50) Same	70% (35/50) Better 30% (15/50) Worse	30% (15/50)
CSF Artifacts	100% (50/50) Same	100% (50/50) Same	100% (50/50)
Motion Artifacts	100% (50/50) Same	100% (50/50) Same	100% (50/50)
Susceptibility Artifacts	100% (50/50) Same	100% (50/50) Same	100% (50/50)
GM/WM Differentiation	100% (50/50) Same	22% (11/50) Better 78% (39/50) Same	78% (39/50)
Image Sharpness	100% (50/50) Same	60% (30/50) Better 40% (20/50) Same	40% (20/50)
Flow Void Visualization	100% (50/50) Same	100% (50/50) Same	100% (50/50)
Extracranial Structure Evaluation	100% (50/50) Same	100% (50/50) Same	100% (50/50)

### 3D T1-weighted SPGR CS = 8

Overall image quality and degree of noise/artifacts were rated excellent or sufficient in 78% (39/50) on 3D T1-weighted SPGR CS = 8 with AI reconstruction, and diagnostic confidence was rated excellent or sufficient in 82% (41/50) (Figs. 2 and 4b). AI reconstruction improved overall image quality, subjective SNR and diagnostic confidence in 94% (47/50), 96% (48/50) and 94% (47/50) of exams when compared with standard CS reconstruction, respectively. However, 3D T1-weighted SPGR CS = 2.5 with AI was felt to be better than 3D T1 SPGR CS = 8 with AI in overall image quality, subjective SNR and diagnostic preference in 88% (44/50), 62% (31/50) and 86% (43/50) of exams, respectively. These and other data are summarized in Tables 2 and 3. Quantitative analyses of signal intensities (mean ROI area = 58.5 ± 11.5 mm^2^) yielded significantly higher aSNR in both the white matter (27.7 ± 8.9 vs. 17.8 ± 6.2, *p* < 0.005 ) and grey matter (20.5 ± 6.2 vs. 13 ± 2.7, *p* < 0.005) in the AI compared to standard CS reconstruction, respectively. The gray-white matter aCNR was also significantly higher in the AI (4.5 ± 1.1) compared to standard CS reconstruction (2.9 ± 0.6, *p* < 0.005).

**Table 2:** T1-weighted SPGR CS = 8 AI vs. standard CS reconstruction

T1 CS 8 AI vs. Standard CS Reconstruction	Reviewer 1 (*n* = 25)	Reviewer 2 (*n* = 25)	% Concordance
Overall Image Quality	96% (24/25) Better 4% (1/25) Same	92% (23/25) Better 8% (2/25) Same	96% (24/25)
Subjective SNR	100% (25/25) Better	92% (23/25) Better 8% (2/25) Same	92% (23/25)
Diagnostic Preference	96% (24/25) Better 4% (1/25) Same	92% (23/25) Better 8% (2/25) Same	96% (24/25)
CSF Artifacts	100% (25/25) Same	100% (25/25) Same	100% (25/25)
Motion Artifacts	100% (25/25) Same	4% (1/25) Better 96% (24/25) Same	96% (24/25)
Susceptibility Artifacts	100% (25/25) Same	100% (25/25) Same	100% (25/25)
GM/WM Differentiation	52% (13/25) Better 48% (12/25) Same	84% (21/25) Better 16% (4/25) Same	60% (15/25)
Image Sharpness	84% (21/25) Better 16% (4/25) Same	92% (23/25) Better 8% (2/25) Same	92% (23/25)
Flow Void Visualization	100% (25/25) Same	24% (6/25) Better 76% (19/25) Same	76% (19/25)
Extracranial Structure Evaluation	40% (10/25) Better 60% (15/25) Same	40% (10/25) Better 60% (15/25) Same	60% (15/25)

**Table 3:** T1-weighted SPGR CS = 2.5 AI reconstruction vs. CS = 8 AI reconstruction

T1 CS 2.5 AI vs. CS 8 AI	Reviewer 1 (*n* = 25)	Reviewer 2 (*n* = 25)	% Concordance
Overall Image Quality	84% (21/25) Better 16% (4/25) Same	92% (23/25) Better 8% (2/25) Worse	84% (21/25)
Subjective SNR	52% (13/25) Better 48% (12/25) Same	72% (18/25) Better 20% (5/25) Same 8% (2/25) Worse	48% (12/25)
Diagnostic Preference	80% (20/25) Better 20% (5/25) Same	92% (23/25) Better 8% (2/25) Worse	80% (20/25)
CSF Artifacts	100% (25/25) Same	100% (25/25) Same	100% (25/25)
Motion Artifacts	16% (4/25) Better 84% (21/25) Same	16% (4/25) Better 84% (21/25) Same	92% (23/25)
Susceptibility Artifacts	4% (1/25) Better 96% (24/25) Same	4% (1/24) Better 96% (24/25) Same	92% (23/25)
GM/WM Differentiation	37.5% (15/25) Better 62.5% (10/25) Same	37.5% (12/25) Better 62.5% (13/25) Same	56% (14/25)
Image Sharpness	72% (18/25) Better 28% (7/25) Same	64% (16/25) Better 28% (7/25) Same 8% (2/25) Worse	68% (17/25)
Flow Void Visualization	12% (3/25) Better 88% (22/25) Same	16% (4/25) Better 81% (21/25) Same	96% (24/25)
Extracranial Structure Evaluation	20% (5/25) Better 80% (20/25) Same	16% (4/25) Better 84% (21/25) Same	96% (24/25)

### 3D T1-weighted SPGR CS = 5

The overall image quality, degree of noise/artifacts and diagnostic confidence were all rated as excellent or sufficient in 91.7% (44/48) of evaluations on 3D T1-weighted SPGR CS = 5 with AI reconstruction (Fig. 3c). AI reconstruction improved overall image quality, subjective SNR, and diagnostic confidence in 100% (46/26) of evaluations when compared with standard CS reconstruction. However, 3D T1-weighted SPGR CS = 2.5 with AI reconstruction was felt to be better than 3D T1 SPGR CS = 5 with AI in overall image quality, subjective SNR, and diagnostic preference in 70.8% (34/48), 45.8% (22/48) and 62.5% (30/48) of exams, respectively. These and other data are summarized in Tables 4 and 5. Quantitative analyses of signal intensities (mean ROI area = 60.9 ± 13.9 mm^2^) yielded significantly higher aSNR in both the white matter (28.7 ± 10.2 vs. 20 ± 4.9, *p* < 0.005) and grey matter (24.4 ± 6.4 vs. 15.5 ± 3.7, *p* < 0.005) in the AI compared to standard CS reconstruction, respectively. The gray-white matter aCNR was also significantly higher in the AI (5 ± 1.8) compared to standard CS (3.3 ± 0.7, *p* < 0.005) reconstruction.

(a-c): Image assessment of 3D T1 SPGR images of the brain with AI reconstruction at CS = 2.5 (a), CS = 8 (b) and CS = 5 (c) by 2 neuroradiologists. Parameters were rated on a 3 point scale (poor, sufficient, excellent) looking at overall image quality (take all imaging facets into account), subjective SNR (assessment of signal intensity versus noise) and diagnostic preference (one’s ability to make the correct diagnosis).

**Table 4:** T1-weighted SPGR CS = 5 AI vs. standard CS reconstruction

T1 CS 5 AI vs. Standard CS Reconstruction	Reviewer 1 (*n* = 23)	Reviewer 2 (*n* = 23)	% Concordance
Overall Image Quality	100% (23/23) Better	100% (23/23) Better	100% (23/23)
Subjective SNR	100% (23/23) Better	100% (23/23) Better	100% (23/23)
Diagnostic Preference	100% (23/23) Better	100% (23/23) Better	100% (23/23)
CSF Artifacts	100% (23/23) Same	100% (23/23) Same	100% (23/23)
Motion Artifacts	100% (23/23) Same	4.3% (1/23) Better 95.7% (22/23) Same	95.7% (22/23)
Susceptibility Artifacts	100% (23/23) Same	100% (23/23) Same	100% (23/23)
GM/WM Differentiation	78.3% (18/23) Better 21.7% (5/23) Same	78.3% (18/23) Better 21.7% (5/23) Same	56.5% (13/23)
Image Sharpness	78.3% (18/23) Better 21.7% (5/23) Same	100% (23/23) Better	78.3% (18/23)
Flow Void Visualization	4.3% (1/23) Better 95.7% (22/23) Same	4.3% (1/23) Better 95.7% (22/23) Same	91.3% (21/23)
Extracranial Structure Evaluation	60.9% (14/23) Better 39.1% (9/23) Same	30.4% (7/23) Better 69.6% (16/23) Same	43.5% (10/23)

**Table 5:** T1-weighted SPGR CS = 2.5 AI reconstruction vs. CS = 5 AI reconstruction

T1 CS 2.5 AI vs. CS 5 AI	Reviewer 1 (*n* = 24)	Reviewer 2 (*n* = 24)	% Concordance
Overall Image Quality	70.8% (17/24) Better 25% (6/24) Same 4.2% (1/24) Worse	70.8% (17/24) Better 29.2% (7/24) Same	70.8% (17/24)
Subjective SNR	45.8% (11/24) Better 54.2% (13/24) Same	45.8% (11/24) Better 54.2% (13/24) Same	75% (18/24)
Diagnostic Preference	54.2% (13/24) Better 45.8% (11/24) Same	70.8% (17/24) Better 29.2% (7/24) Same	75% (18/24)
CSF Artifacts	4.2% (1/24) Better 95.8% (23/24) Same	100% (24/24) Same	95.8% (23/24)
Motion Artifacts	29.2% (7/24) Better 66.7% (16/24) Same 4.2% (1/24) Worse	8.3% (2/24) Better 87.5% (21/24) Same 4.2% (1/24) Worse	75% (18/24)
Susceptibility Artifacts	4.2% (1/24) Better 95.8% (23/24) Same	4.2% (1/24) Better 95.8% (23/24) Same	91.7% (22/24)
GM/WM Differentiation	37.5% (9/24) Better 62.5% (15/24) Same	37.5% (9/24) Better 62.5% (15/24) Same	75% (18/24)
Image Sharpness	62.5% (15/24) Better 37.5% (9/24) Same	62.5% (15/24) Better 37.5% (9/24) Same	60.9% (14/24)
Flow Void Visualization	16.7% (4/24) Better 83.3% (20/24) Same	4.2% (1/24) Better 95.8% (23/24) Same	87.5% (21/24)
Extracranial Structure Evaluation	66.7% (16/24) Better 75% (18/24) Same	8.3% (2/24) Better 91.7% (22/24) Same	75% (18/24)

## Discussion

In this study we observed that in the majority of patients evaluated, AI reconstruction improved the overall image quality of highly accelerated (CS = 5 and 8) 3D T1-weighted SPGR images. Quantitative analysis of gray and white matter aSNR and grey-white matter aCNR also revealed significant improvement with AI compared with standard CS reconstruction in both the CS = 5 and CS = 8 groups. While the AI reconstructed images in the CS = 5 and CS = 8 groups had excellent or sufficient overall image quality in the majority of patients, CS = 2.5 with AI still had better image quality than accelerated compressed sense (CS = 5 and 8) with AI in most cases. Also compared with standard CS reconstruction, AI reconstruction demonstrated the same overall image quality in majority of evaluations and demonstrated no statistically significant differences in quantitative analysis of aSNR and grey-white matter aCNR for the clinical sequence of CS = 2.5. Among the types of artifacts examined, AI reformats did not appear to exacerbate these artifacts in this study population when compared to standard CS reconstruction.

The use of AI algorithms for under-sampled k-space data has been described as a potential method to decrease scan time without compromising image quality in proton density of the knee, 2D fast spin echo (FSE) of the hip and shoulder, and 3D T2 turbo-spin echo (TSE) of the lumbar spine MRI [5, 7–9]. Improved image quality with AI reconstruction algorithms in diffusion weighted imaging (DWI) of the head, neck, and pancreas, and T2-TSE of the prostate have also been described [10–13]. There are also multiple studies describing its use in cardiac imaging including on black blood T2-weighted images, late gadolinium enhancement image quality, and evidence of preserved biometric data marked by biventricular volumetric indices on cine cardiac MRI [6, 14–16].

There is a growing body of literature examining the use of AI-based denoising reconstruction of MRI in the adult brain [17, 18]. In addition, there is an increasing volume of literature describing its potential uses in clinical practice, including ultra-fast imaging, oncology patients and those with Alzheimer’s disease [19–22]. Similar clinical literature in the pediatric brain, however, is sparse. Examples include recent literature describing the use of AI reconstruction to reduce scan time for synthetic MRI and improve imaging quality for T2-weighted MRI of the pediatric brain [23, 24]. Our study adds to this literature by exploring the use of AI reconstruction for accelerated 3D T1-weighted SPGR images. Notably, sagittal T1-weighted imaging is a recommended component of a routine clinical pediatric brain MRI protocol which in practice, is often obtained through the use of 3D T1-weighted SPGR sequences [25]. Through the evaluation of CS at 3 different levels, our study demonstrates how AI reconstruction techniques can be applied to enable appreciably shorter scan times [26]. MRI of the pediatric brain is the most frequently performed MRI exam in our department and methods allowing for decreased scan time with preserved image quality will be valuable in improving work flow efficiency, patient throughput, scheduling access and ultimately patient satisfaction in the future.

The strengths of our study include its prospective nature, a relatively large study population, consistent scan parameters on a single clinical magnet, and the systematic review of the images by 2 experienced pediatric neuroradiologists for potential use in clinical practice. Our study, however, does have some limitations. The lack of blinding of the radiologists to the sequence that was being viewed when comparing images can potentially introduce bias, though was difficult to avoid given the study radiologists’ knowledge and experience with the clinical protocol. Also, the number of patients in each CS group was relatively small, and the ability to evaluate a broader range of CS values in more patients would have been informative. In addition, the specific exclusion criteria implemented resulted in a relatively high percentage of normal exams. Thus, the patient population evaluated may not be representative of a broader clinical pediatric practice. Finally, the single scanner, single-institutional nature of this study also limits the generalizability of the results to other field strengths, MRI vendors and institutions.

This study examines the image quality of AI reconstruction of accelerated 3D T1 SPGR images of the pediatric brain in our institution in a relatively large number of patients using an AI reconstruction algorithm. We have shown that AI reconstruction improves overall image quality and SNR, both qualitative and quantitative, in accelerated (CS = 5 and 8) 3D T1W SPGR images in the majority of pediatric patients. We conclude that the integration of AI reconstruction for this sequence into routine clinical practice in order to reduce scan acquisition time is likely safe and feasible.

## Electronic supplementary material

Below is the link to the electronic supplementary material.

Supplementary Material 1
