# Supplementary material for: Evaluation of 3D T1-weighted spoiled gradient echo MR image quality using artificial intelligence image reconstruction techniques in the pediatric brain
Source: Neuroradiology. 2024 Jul 5;66(10):1849–57. doi: 10.1007/s00234-024-03417-9 (PMC11424660; doi:10.1007/s00234-024-03417-9)
Supplement: Supplementary file 1 — Supplementary Material 1 [file 234_2024_3417_MOESM1_ESM.docx]

**Supplementary Table A: Description of Parameters Examined**

| Imaging Parameter | Description |
| --- | --- |
| Overall Image Quality | Assessment of which images look better overall taking all imaging facets into account. |
| Subjective SNR | Subjective assessment of the quality of image signal intensity compared to image noise on a given sequence. |
| Diagnostic Preference | Assessment of one’s ability to make the correct diagnosis given the quality of a given sequence. |
| CSF Artifacts | Evaluation of flow artifacts within the CSF (ventricles, cisterns, extra-axial CSF spaces). |
| Motion Artifacts | Assessment of the degree of patient motion degrading the image quality. |
| Susceptibility Artifacts | Degree of signal loss associated with areas of magnetic susceptibility, mainly air in mastoid air cells and paranasal sinuses |
| Grey-White Matter Differentiation | Evaluation of one’s ability to accurately differentiate grey and white matter both in overall signal intensity and along their interfaces. |
| Image Sharpness | Subjective evaluation of spatial resolution of the intracranial structures. |
| Flow Void Visualization | Ability to accurately evaluate the major arterial flow voids (mainly cavernous internal carotid arteries on FLAIR images). |
| Extra-Cranial Structure Visualization | Evaluation of quality of extra-calvarial soft tissues such as the scalp and orbits. |
